# Disease-resistant varieties of Chinese cabbage (*Brassica rapa* L. ssp. *pekinensis*) inhibit *Plasmodiophora brassicae* infestation by stabilising root flora structure

**DOI:** 10.3389/fpls.2024.1328845

**Published:** 2024-03-05

**Authors:** Tianyi Fang, Xueyu Han, Yanling Yue

**Affiliations:** College of Landscape and Horticulture, Yunnan Agricultural University, Kunming, China

**Keywords:** *Plasmodiophora brassicae*, 16S rRNA, root microbiota, Chinese cabbage, exudates

## Abstract

The application of disease-resistant varieties is the most cost-effective method for solving the problem of clubroot. “Shangpin,” a disease-resistant variety of Chinese cabbage with broad-spectrum immunity to *Plasmodiophora brassicae* (*P. brassicae*), was screened in a previous study. Based on 16S rRNA sequencing technology, we annotated the compositional differences between the rhizosphere, rhizoplane, and endosphere bacterial communities of “Shangpin” and “83-1” under *P. brassicae* stress. Alpha diversity analysis showed that the abundance of microorganisms in the root system of “83-1” changed more than that of “Shangpin” after *P. brassicae* infestation, and Beta diversity analysis indicated that *Flavobacterium* and *Sphingomonas* may mediate clubroot resistance, while *Nitrospira*, *Nitrosospira*, and *Pseudomonas* may mediate *P. brassicae* infestation among the bacteria in the Top 10 abundances. Microbial functional analyses showed that the root microorganisms of “83-1” were metabolically weakened after *P. brassicae* inoculation and were inhibited in competition with pathogenic bacteria. Conversely, the root microorganisms of “Shangpin” maintained the strength of their metabolic capacity, which took a favorable position in competition with the pathogen and inhibited the growth and development of the pathogen, thus showing resistance. Root secretions of “Shangpin” significantly inhibited the incidence and disease index of clubroot, which indicated that under clubroot stress, resistant varieties maintain root microbial diversity and microbial community functions through specific root exudates, enriching the genera *Flavobacterium* and *Sphingomonas*, thus showing resistance. The results of this study reveal the resistance mechanism of resistant varieties to clubroot and provide new insights into the prevention and control of clubroot in Chinese cabbage.

## Introduction

Chinese cabbage (*Brassica rapa subsp. pekinensis*) is native to China. It is an important vegetable crop of the genus Brassica in the family Brassicaceae and plays an important role in human diet. Chinese cabbage is widely cultivated, and its planting area accounts for 4.22% of the world’s total vegetable area. In addition to potatoes, it is second only to tomatoes, onions, and peas. China’s vegetables account for 40.09% of the world’s total area (FAO data, 2021)^1^, of which Chinese cabbage accounts for approximately 15%, with a production of 160 million tons and an output value of more than 60 billion yuan (Liu et al., 2024). However, clubroot attacks have dealt serious blows to Chinese cabbage production. Clubroot, known as the cancer of cruciferous crops, is a worldwide soil-borne disease caused by *Plasmodiophora brassicae*, and it specifically infects cruciferous plants. This pathogen invades the root cortex, stimulates the reproduction and division of cortical cells, causes root swelling and the formation of tumors of different sizes (Sang et al., 2009), and hinders the absorption of water and nutrients by the root system (Dixon, 2009), causing wilting of the aboveground parts, and the Chinese cabbage cannot form leaf head with commercial value. By 2022, more than 80 countries had reported the existence of clubroot disease. The disease continues to spread at an alarming rate, causing significant economic losses (Javed et al., 2023). In some areas, lettuce and other crops must be planted instead because of the inability to solve the problem of clubroot, which greatly limits the sustainable production of Chinese cabbage.

The occurrence of soil-borne diseases is the result of interactions between host plants, pathogens, and the rhizosphere environment. If the rhizosphere is conducive to the occurrence and infection of pathogens, it can cause diseases. Conversely, suppose the rhizosphere environment is conducive to the growth and development of plant roots. In that case, it will control the occurrence of diseases but will not be conducive to the occurrence of pathogens. Microbial composition is an important factor affecting the plant rhizosphere environment, and it plays a vital role in promoting plant growth and development and in responding to biotic and abiotic stresses (Zhou et al., 2022; Yuan et al., 2018; Hu et al., 2018; Hu et al., 2018). The function of the microbial community is closely related to its regulation by the host. Under specific stresses, plants form specific microbial communities that can improve their resistance to the corresponding stress (Bai et al.,2022). Tomato roots secrete malic acid to enrich *Bacillus* subtilis and inhibit *Pseudomonas* syringae pathovar tomato (Pst), thereby enhancing plant resistance (Rudrappa et al., 2008). Berendsen et al. (2018) found that *Arabidopsis* continuously enriches beneficial bacteria, such as *Xanthomonas* and *Stenotrophomonas*, to improve plant resistance against downy mildew, and this resistance can even be extended to the next generation (Raaijmakers and Mazzola, 2016). Another study found that watermelon roots enriched with *Bacillus* amyloides L3 produced volatile substances with strong fungal resistance (2-nonanone, 2-heptanone, etc.) to inhibit the occurrence of Fusarium wilt (Wu et al., 2019). It has also been found that maize root secretions are enriched with Oxalobacteraceae to enhance maize nitrogen acquisition for plant resistance (Yu et al., 2021). In summary, plant roots screen out microbiomes that are beneficial to them through special secretions (Zhang et al., 2019). These beneficial microorganisms help defend against pathogenic microorganisms while simultaneously inducing the expression of plant defense genes to mitigate the negative effects of pathogenic microorganisms (Bukhat et al., 2020; Korenblum et al., 2020; Enagbonma et al., 2023).

Han et al. (2021) identified the resistance of 21 disease resistant varieties to Clubrot in 34 regions in Yunnan Province, China. The only variety, ‘Shangpin,’ with broad-spectrum immune properties, was screened out, and it showed immunity to 34 species of *P. brassicae*. This shows that it is an excellent antisource material. However, it remains unclear whether there is any difference in the structure of microorganism in the rhizosphere, rhizoplane, and endosphere of susceptible varieties and whether these differences are related to disease resistance. The mechanisms used to achieve disease resistance are also unknown.

To understand the disease resistance mechanism of ‘Shangpin,’ a clubroot-resistant variety of *Brassica rapa*, we sequenced the 16S rRNA of microorganisms from the rhizosphere, rhizoplane, and endosphere of ‘Shangpin’ and a susceptible variety ‘83-1,’ with or without *P. brassicae* inoculation. We compared the root microbial flora structures of the two varieties and analyzed how they influenced the disease resistance of ‘Shangpin.’ This study offers valuable insights for biological control of clubroot.

## Materials and methods

### Plant materials

Chinese cabbage varieties ‘83-1’ and ‘Shangpin’ are respectively susceptible and immune to Clubroot in 34 regions in Yunnan Province, China (Han et al., 2021). Their disease resistance is shown in **Supplementary Figure 1**.

### Clubroot fungus

Collected from Shaqiao Town, Nanhua County, Chuxiong Prefecture, Yunnan Province, and identified by the laboratory as Williams No. 4 pathogenic type, which is the most virulent race in 34 places (Han et al., 2021).

### Experimental treatment

There are four treatments in the experiment, Rc (‘Shangpin’, not inoculated with *P. brassicae*), R1 (‘Shangpin’, inoculated with *P.brassicae*), Sc (‘83-1’, not inoculated with *P. brassicae*), S1 (‘83-1’, inoculated with *P.brassicae*). The control was a blank soil sample.

### Inoculation of *Plasmodiophora brassicae*

In order to avoid differences in factors such as bacterial load, soil fertility, weeds, and microbial composition in field sampling, blank soil samples were used in pots to inoculate bacteria in this experiment. Blank soil sample: collected from the vegetable base field of Yunnan Agricultural University, the previous crop was Chinese cabbage. Pass through a 100-mesh sieve (Lu et al., 2015), randomly select 5 tubes (50ml) as blank controls (CK), and store in a -80°C refrigerator for later use. Refer to the method of Chen et al. (2016) to extract dormant spores of *P.brassicae*, suspend them in 5% sucrose solution, and count them with a hemocytometer. Peat is sieved, and mixed evenly with dormant spore suspension and sterile water to prepare *P. brassicae* inoculated soil. The concentration of dormant spores in *P. brassicae* inoculated soil is 1×10^7^/g. The bacterial soil was activated in the dark at 37°C for 72 hours and set aside. Take a 23cm diameter plastic bowl and put 4L of blank soil sample in it. Make a hole 1cm in diameter and 2cm deep in the middle, and add 1g of *P. brassicae* inoculated soil into the hole. Sow 1 germination seed on the *P. brassicae* inoculated soil and cover it with a blank soil sample. 72 pots per treatment, Hoagland nutrient solution administered.

### Collection of root microorganisms

60 days after inoculated *P.brassicae*, 40 pots of plants from each treatment were randomly selected. First use a sterilized knife to gently remove the topsoil from the nutrient bowl. Slowly remove the root system of the plant, shake off the large pieces of soil around the root system, and retain about 1mm of rhizosphere soil on the root surface of the plant. Every 8 plants were mixed and sieved, and put into a 50ml test tube as a rhizosphere microbial soil sample; the roots of the 8 plants were first vibrated vigorously in a vortex oscillator for 15 seconds, and then placed in PBS for ultrasonic vibration (50-60Hz) for approx 1min. Vortex and ultrasonic vibrate twice, then centrifuge at 3400 rpm for 10 min, discard the supernatant, and resuspend the precipitated microorganisms (Howard et al., 2017) as a rhizoplane microbial sample; then treat the root system with 75% alcohol. 30s, then surface disinfected with 2% sodium hypochlorite for 15min, rinsed 3 times with sterile water, and the drained roots were used as endosphere microbial samples. Each rhizosphere, root surface, and root sample was taken five times.

### Identification of disease resistance phenotypes

When sampling, investigate the incidence rate (the percentage of diseased plants in the total number of plants surveyed) and disease index. According to the grading standards for clubroot diseases in the seedling stage of cruciferous crops, the disease is divided into six levels (Hou and Cai, 2014): level 0 if there are no tumors on the roots; level 1 if there are small tumors on the lateral roots. The main root is swollen and its diameter is less than 2 times that of the stem base, which is grade 3. The main root is swollen and its diameter is 2-3 times that of the base of the stem, which is grade 5. The main root is swollen and its diameter is 3-4 times that of the base of the stem, which is grade 7. The main root is swollen and its diameter is more than 4 times that of the base of the stem, which is grade 9.


$$\text{Diease index}=\frac{\sum \left(\text{Number of plants at each level of the disease}\times \text{relative magnitude value}\right)}{\text{Total number of plants surveyed}\times 9}\times 100$$

### 16S rRNA sequencing

Total DNA was extracted using the PowerSoil DNA extraction method. DNA purity and concentration were checked by agarose gel electrophoresis. The V3-V4 variable region of the bacterial 16S rRNA gene was amplified using specific primers (Nossa, 2010) 343F (5’-TACGGRAGGCAGCAGCAG-3’) and 798R (5’-AGGTATCTAATCCT-3’). PCR electrophoresis was performed using Takara Ex Taq hi-fi enzyme (from Takara Corporation). After detection, magnetic beads are used for purification, and after purification, it is used as a second-round PCR template for amplification. Electrophoresis detection was used again. After detection, magnetic beads were used for purification. After purification, the PCR product was quantified using Qubit 6. Mix equal amounts of samples according to the concentration of PCR products and perform Illumina MiSeq sequencing.

### Illumina RNA-seq sequencing

The library was sequenced using an Illumina HiSeq 2000 instrument with paired-end 100-bp reads. To obtain clean reads, we removed dirty reads containing adapters and unknown bases (N > 5%) as well as low-quality bases (scores<20) from the raw data. The raw sequence data reported in this paper have been deposited in the Genome Sequence Archive at the BIG Data Center under the accession number PRJCA017453.

### Collection of root exudates and root irrigation

‘Shangpin’ and ‘83-1’ were germinated and the seedlings were floated in 136 holes. The substrate was mixed with peat, perlite, and vermiculite in a volume ratio of 2:1:1, and treated with and without inoculation of *P.brassicae*. The inoculation method was the same as above. Each treatment has a separate flotation tank and Hoagland nutrient solution management. Collect the nutrient solution containing root exudates every 3 days, filter and centrifuge, and take the supernatant. They were recorded as RcE (root exudates not inoculated with *P.brassicae* ‘Shangpin’), R1E (root exudates inoculated with *P.brassicae* ‘Shangpin’), ScE (root exudates not inoculated with *P.brassicae* ‘83-1’), S1E (Root exudates inoculated with *P.brassicae* ‘83-1’). Replace with new nutrient solution at the same time.

Fill a nutrient bowl with a diameter of 9 cm with substrate (same as above), sow ‘83-1’ for germination, and inoculate the *P.brassicae* (method as above). The roots were irrigated with 4 types of root exudates every 3 days, 50ml per pot. The same volume of water was used as a control. Each treatment included 30 strains and was repeated three times. The disease severity was graded according to the rhizobia disease classification standards of cruciferous crops at the seedling stage, and the disease index was calculated.

### Data analysis

The quality sequences with valid tags obtained from the quality control were classified as OTUs using Vsearch (version 2.4.2) (Rognes et al., 2016) software, with a 97% similarity threshold. The representative sequence with the highest abundance in each OTU was selected. The RDP classifier Naive Bayesian classification algorithm (Wang et al., 2007) was then used to annotate the representative sequences against the database, providing annotation information for the OTUs. An abundance matrix file of OTUs in each sample was constructed based on the number of sequences contained in each OTU. The pynast (v0.1) (Caporaso et al., 2009) software was used to classify the representative sequences of OTUs by sequence comparison, considering sequences with a similarity of 97% or higher as one OTU unit. All representative sequences were annotated against the database, and the annotation results with intervals greater than 0.7 were retained. Finally, the OTU types, annotation information, and representative sequences were counted.

The alpha diversity index was calculated using algorithms such as Kruskal and Wilcoxon. PCA analysis was performed based on the Bray Curtis distance matrix algorithm. Additionally, the significance of differences in the distribution of the sample communities between groups was assessed using p-values for Adonis and Anosim analyses, which were also based on the Bray Curtis distance matrix algorithm. The community structure histogram, heat map, and correlation analysis plots were generated in R.

## Results

**Figure 1:**
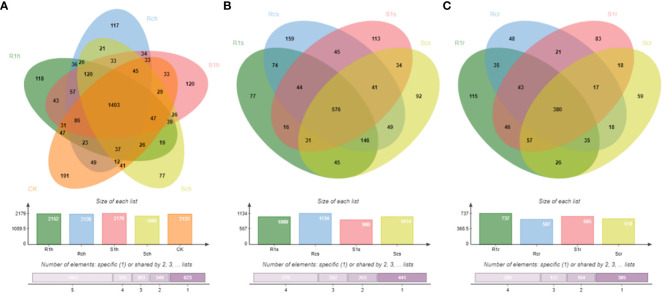
Distribution of OTU in root system. **(A)** Distribution of OTU quantity in rhizosphere bacteria; **(B)** Distribution of OTU quantity in rhizoplane bacteria; **(C)** Distribution of OTU quantity in endosphere bacteria.

Sequencing results of the 16S amplicons showed that a total of 7,863 operational taxonomic units (OTUs), 59,667 clean tags, and 52,148 valid tags were measured in 65 samples (**Supplementary Table 1**). The blank soil sample CK had 2,133 OTUs; the rhizosphere samples of the inoculated resistant variety R1h, the non-inoculated resistant variety Rch, the inoculated susceptible variety S1h, and the non-inoculated susceptible variety Sch had 2,152, 2,126, 2,179, and 1,995 OTUs, respectively. The co-ownership of OTUs in CK, R1h, Rch, S1h, and Sch was 1,403 (**Figure 1A**).

The inoculated resistant and susceptible varieties had 1009 and 900 rhizoplane OTUs, respectively, while the non-inoculated resistant and susceptible varieties had 1134 and 1014 OTUs, respectively. The co-ownership of all four rhizoplanes had 576 OTUs (**Figure 1B**).

In the endosphere of the inoculated resistant variety, 737 OTUs were identified for R1r and 597 OTUs were identified for Rcr in the non-inoculated resistant variety. The inoculated susceptible variety had 665 OTUs for S1r, and the non-inoculated susceptible variety had 610 OTUs for Scr. Three hundred and eighty OTUs were shared by all four varieties (**Figure 1**).

### Alpha diversity analysis

**Figure 2:**
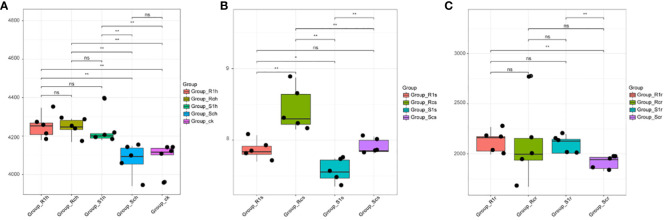
Analysis of a diversity of root bacteria.:R1, RC, S1 and SC were resistant strains with *P.brassicae*, resistant strains without *P.brassicae*, susceptible strains with *P.brassicae* and susceptible strains without *P.brassicae* respectively. From **(A–C)** were rhizosphere, rhizoplane and endophytic bacteria. ns indicates not significant, * indicates significant difference, P value is less than 0.05; ** means very significant difference, P value is less than 0.01.

Alpha diversity analysis of bacterial distribution in the three root compartments (**Figure 2**) revealed that the highest bacterial diversity was observed in the rhizosphere group of the susceptible non-inoculated group Sch. This difference in bacterial diversity was highly significant (P< 0.01) compared to that in the susceptible group S1h after inoculation, the resistant non-inoculated group Rch, and the resistant inoculated group R1h (**Figure 2A**). These findings indicate that both invasion by *P. brassicae* and cultivar resistance can affect rhizosphere bacterial diversity. In the rhizoplane group, there were highly significant differences in bacterial diversity between the susceptible non-inoculated group Scs and the susceptible infected group S1s, as well as the resistant non-inoculated group Rcs (P< 0.01). However, these differences were not significantly different from the resistant-infected group R1s (**Figure 2B**). This suggests that the initial bacterial diversity in the resistant and susceptible varieties of *P. brassicae* differed. However, with the invasion of *P. brassicae*, the bacterial diversity of *P. brassicae* of the resistant and susceptible varieties underwent a shift, where the bacterial diversity of the resistant variety gradually became similar to that of the susceptible non-receptive variety. In the endospheric group, there was no significant difference in bacterial diversity between the susceptible non-inoculated Scr group and the resistant non-inoculated Rcr group. After inoculation with *P. brassicae*, the difference in bacterial diversity between S1r and Scr in the susceptible group was highly significant (P< 0.01), whereas the difference between R1r and Rcr in the resistant group was not significant (**Figure 2C**). These findings suggest that inoculation with *P. brassicae* affects bacterial diversity in the endospheres of susceptible species, but not in resistant species.

**Figure 3:**
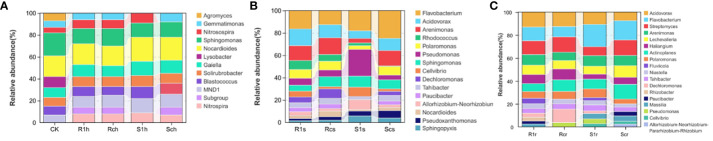
Distribution of rhizosphere, rhizoplane and endosphere bacterial community structures.

​ To analyze the community structure composition of rhizosphere bacteria in different treatments, we examined bacterial genera with a relative abundance greater than 1% (**Figure 3**). The results revealed that CK soil, *Agromyces*, and *Lysobacter* were not present in either the susceptible group or the resistant group among the bacterial genera, with a relative abundance greater than 1% in the rhizosphere. *Nitrosospira* and *Blastococcus* in the Sch group exhibited significantly lower abundances and were not the dominant genera. Additionally, *Gemmatimonas* was previously not the dominant genus of bacteria in the S1h group. For *P. brassicae*, a significant increase in the number of bacterial species was observed. Among them, *Arenimonas* and *Dechloromanas* showed a significant decrease in abundance after inoculation in susceptible varieties but remained relatively stable in resistant varieties. This suggests a possible relationship between these two genera and disease resistance. Conversely, *Pseudomonas* and *Rhizobium* showed a significant increase in abundance after inoculation with susceptible strains, indicating a potential association with susceptibility to Rhizobium. In the endosphere, *Fluvicola* exhibited a significant increase in abundance after inoculation with both susceptible and resistant strains, suggesting a potential relationship with *P. brassicae*. Furthermore, *Paucibacter* decreased in abundance after inoculation in susceptible strains but increased in abundance after inoculation in resistant strains, indicating a possible association with disease resistance.

We compared the number of different bacteria in the rhizosphere, rhizoplane, and endosphere, specifically comparing R1 and S1, R1 and Rc, Rc and Sc, and S1 and Sc. In the rhizosphere soil (**Supplementary Table 2**), the four groups accounted for 6.29%, 5.85%, 7.42%, 7.84%, and 1.8%, 0.88%, 1.43%, and 2.61% of the total, respectively, with significant (P< 0.05) and highly significant (P< 0.01) differences. In the rhizoplane group (**Supplementary Table 3**), the four groups differed significantly (P< 0.05) and highly significantly (P< 0.01), representing 21.24%, 7.75%, 15.44%, 23.92%, and 11.71%, 1.29%, 5.54%, and 12.23%, respectively. Among the endosphere groups (**Supplementary Table 4**), the four groups also showed significant (P< 0.05) and highly significant (P< 0.01) differences, accounting for 4.73%, 4.78%, 6.64%, 8.94% and 1.89%, 1.29%, 1.11%, and 2.66% of the total, respectively. The results from the three root chambers were consistent. The least abundant differential bacteria were found in R1 and Rc at a high significance level, whereas the most abundant bacteria were found in S1 and Sc.

**Figure 4:**
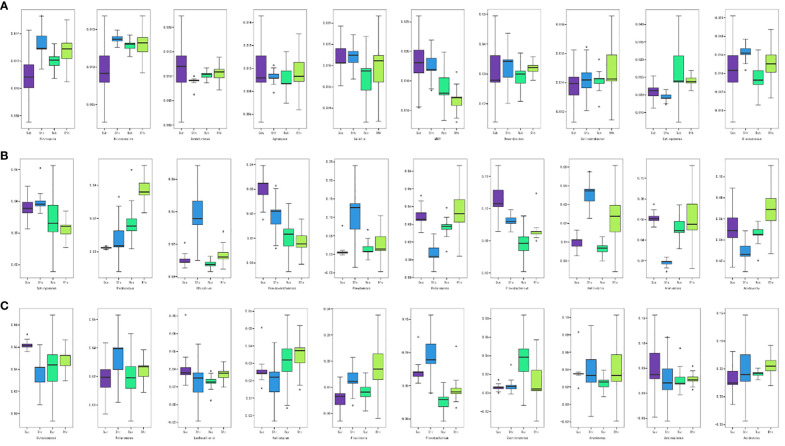
The top 10 different bacteria in **(A)** rhizosphere, **(B)** rhizoplane and **(C)** endosphere.

We conducted a boxplot analysis to examine the Top 10 differential bacterial abundances based on relative abundance. This analysis allowed us to compare the abundance of the dominant differential species within and between groups. Statistical analyses were performed at all levels of phylum, family, genus, and species. The figure presented below (**Figure 4**) focuses on the rhizosphere, rhizoplane, and endosphere Top 10 differential bacteria at the genus level. Our results revealed that there were no significantly dominant genera at the rhizosphere level, but there were significant differences between the *Nitrospira* and *Nitrosospira-*susceptible varieties (**Figure 4A**). This suggests that bacteria from these two genera were enriched after the *P. brassicae* invasion. In the rhizoplane, the abundance of *Pseudomonas* and *Flavobacterium* was significantly higher than that of the other genera (**Figure 4B**). Specifically, the abundance of *Pseudomonas* increased significantly after inoculation of susceptible varieties, indicating its potential association with clubroot infestation. Conversely, *Flavobacterium* decreased in abundance in susceptible varieties but increased in resistant varieties after inoculation with *P. brassicae*, possibly mediating clubroot resistance. *Rhodococcus* and *Acidovorax* are also worth noting, as both genera significantly increased in the resistant varieties after inoculation. Flavobacterium was the dominant genus in the endosphere, but unlike the changes in abundance observed in the rhizoplane (**Figure 4C**), this genus increased in abundance in susceptible varieties after inoculation, likely originating from the rhizoplane in the root. Overall, the abundance of most genera remained relatively stable before and after inoculation of resistant varieties, whereas the abundance of genera in susceptible varieties fluctuated considerably. This confirms our hypothesis that disease-resistant varieties demonstrate resistance by stabilizing the structure of the microbial community in the root system.

### Beta diversity analysis

**Figure 5:**
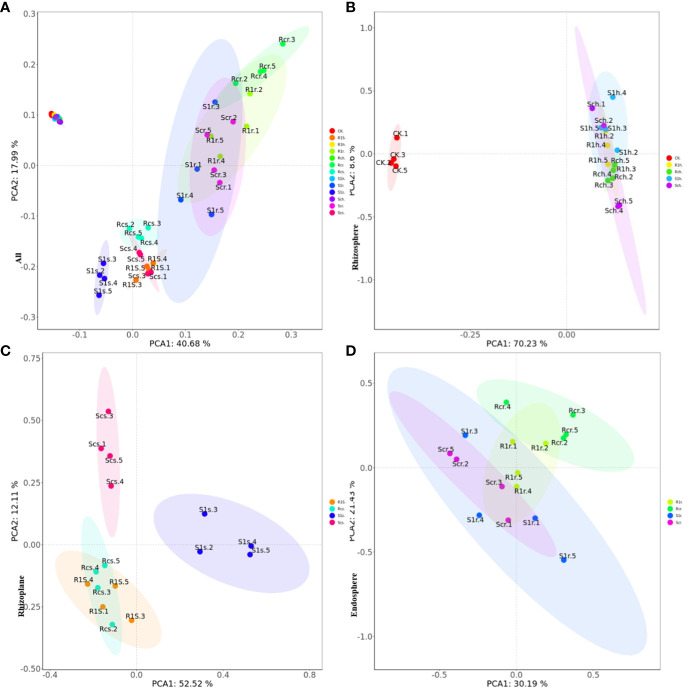
PCA principal component analysis showing the effect of *P. brassicae* on the microbial community structure of **(A)** root, **(B)** rhizosphere, **(C)** rhizoplane and **(D)** endosphere.

The results of principal component analysis (PCA) indicated that there were significant differences between rhizosphere bacteria and rhizoplane and endosphere bacteria (**Figure 5A**). Additionally, the changes in rhizosphere bacteria were not significant, regardless of *P. brassicae* inoculation (**Figure 5B**). This suggests that the effect of cabbage on rhizosphere microorganisms after inoculation was minimal. Furthermore, the treatment of rhizoplane bacterial resistance showed significant differences compared with the susceptible treatment (**Figure 5C**). Although there were no significant differences in bacterial composition between the treatments of resistant varieties, notable differences were observed in the treatments of susceptible varieties. This indicates that the bacterial composition of the rhizoplane varies among different plant varieties, and susceptible cabbages experience significant changes in rhizoplane bacteria after infestation with *P. brassicae*. However, resistant varieties can resist this effect, which may be attributed to their disease resistance mechanisms. There were no significant differences between treatments in the endosphere (**Figure 5D**), indicating that the development of *P. brassicae* did not significantly affect the bacterial composition of the endosphere.

### Correlation between bacteria in three root chambers

**Figure 6:**
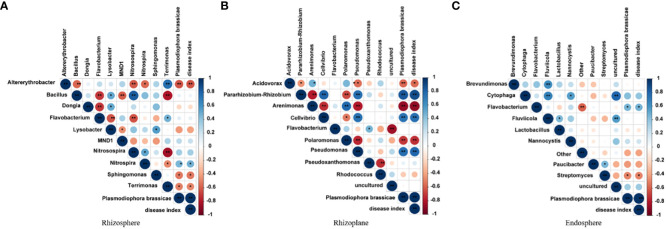
Correlation analysis of bacteria in **(A)** rhizosphere, **(B)** rhizoplane and **(C)** endosphere. * indicates significant difference, P value is less than 0.05; ** meansvery significant difference, P value is less than 0.01.

Bacterial correlation studies revealed stronger correlations between the rhizosphere and rhizoplane bacteria, particularly the rhizoplane bacteria (**Figure 6**). However, no significant correlation was observed for the endosphere bacteria. In the correlation analysis, *P. brassicae* showed a positive correlation with the disease index. In the rhizosphere, *Altererythrobacter* exhibited a negative correlation with *P. brassicae* and the disease index (P< 0.01), while *Nitrospira* exhibited a positive correlation with *P. brassicae* and the disease index (P< 0.05). *Sphingomonas* and *Terrimonas* were both negatively correlated with *P. brassicae* and the disease index (P< 0.05). Among the rhizoplane bacteria, *Arenimonas*, *Polaromonas*, and *Pseudomonas*, all three bacterial genera, were significantly positively correlated with *P. brassicae* and the disease index (P< 0.01). In contrast, *P. brassicae* and *Cellvibrio* were significantly negatively correlated (P< 0.01). Among endosphere bacteria, *Flavobacterium* and *Streptomyces* showed a significant negative correlation (P< 0.05) with *P. brassicae* and the disease index. These findings suggest that rhizoplane bacteria are more susceptible to the influence of *P. brassicae*, which consequently affects the disease index.

### Phylogenetic evolutionary trees and species abundance

**Figure 7:**
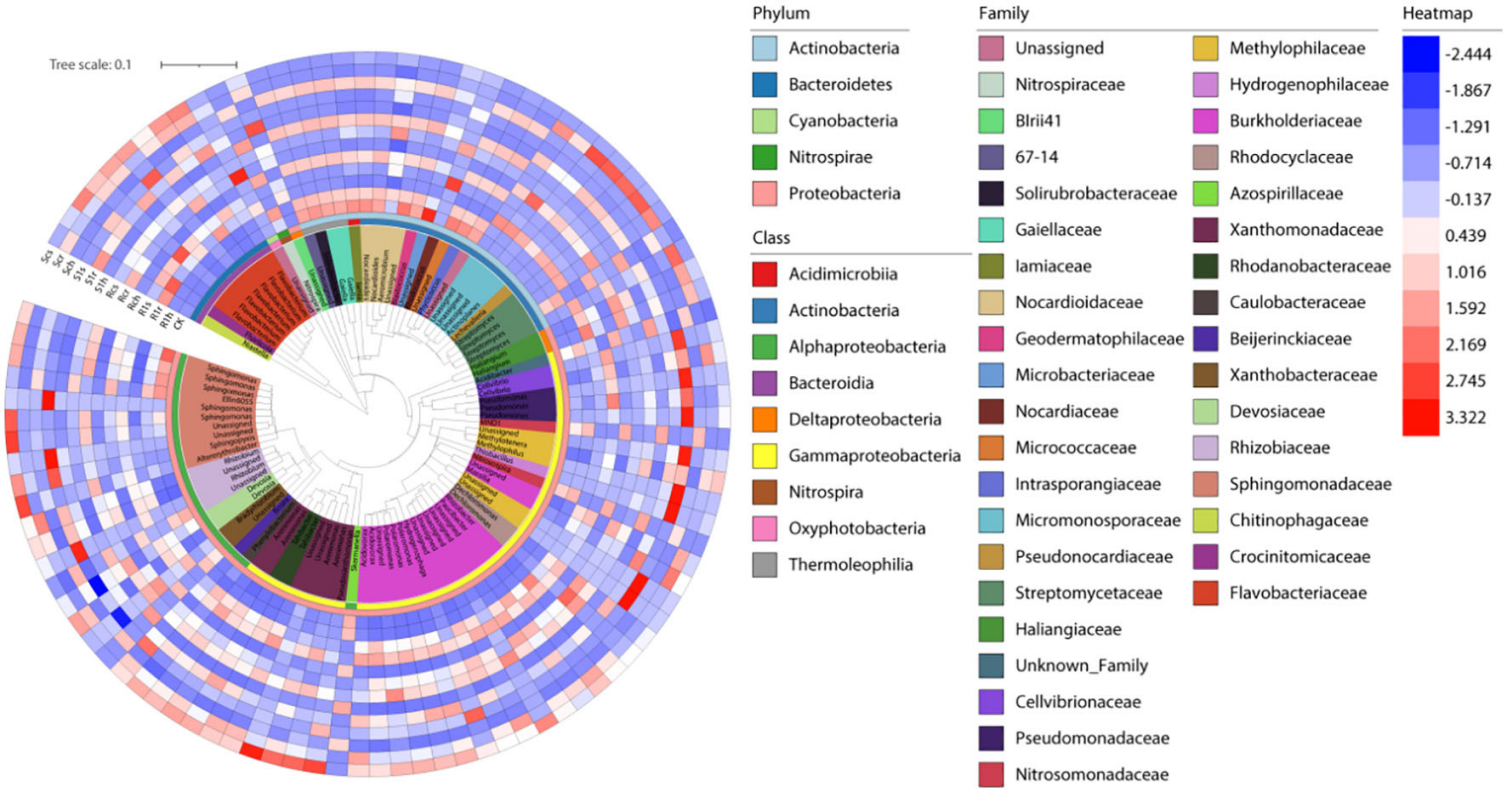
Evolution and classification of bacteria with relative abundance in the top 100.

We constructed an evolutionary tree with heat maps for 13 groups to analyze the relative abundance of the Top 100 bacteria at the genus level (**Figure 7**). These 100 bacterial genera were distributed across five phyla, seven classes, and 37 families. The relative abundance of bacteria on the rhizoplane in infected varieties affected by clubroot fungi was either extremely high or extremely low. For example, bacteria such as *Rhizobium* and *Sphingomonas* showed significant variation. This suggests that rhizoplane bacteria of susceptible plants are more vulnerable to plant or foreign pathogenic bacteria. In contrast, rhizosphere and endophytic bacteria were less affected by infection. The changes in bacterial composition in disease-resistant varieties were not as prominent, indicating that external factors influenced them less. The rhizosphere bacteria of the resistant varieties were similar to those of the control group (CK), indicating that they were less affected (**Supplementary Figure 1**). This explains why the resistant varieties do not exhibit symptoms.

### Effect of *P.brassicae* on the microbial function of three root compartments

**Figure 8:**
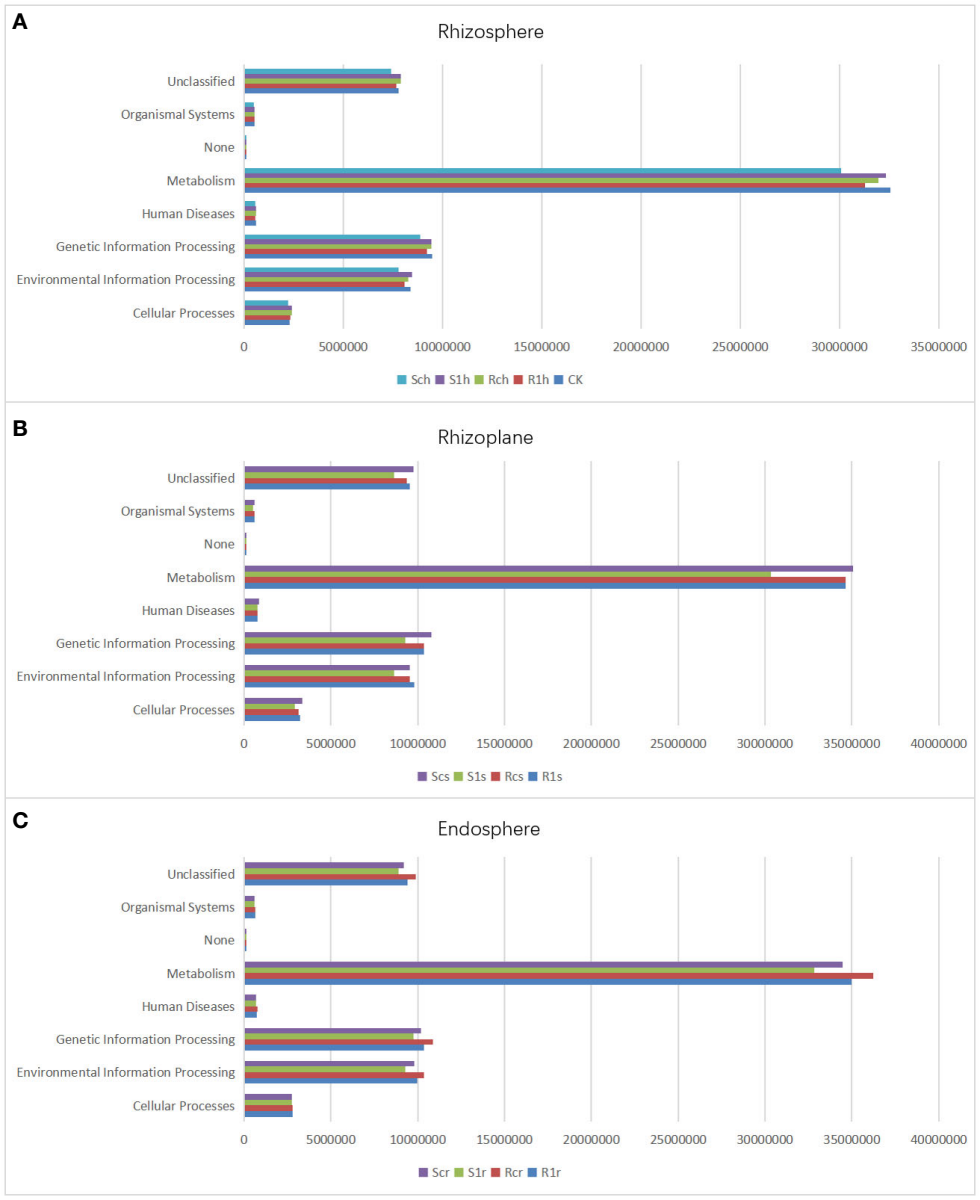
KEGG differential pathway in **(A)** rhizosphere, **(B)** rhizoplane and **(C)** endosphere.

To investigate the functional changes in different resistant cabbage microorganisms under *P. brassicae* infestation conditions, we performed functional prediction of gene information using PICRUSt software. Our analysis included 36 libraries of rhizosphere, rhizoplane, and endosphere bacteria from Chinese cabbage. The results showed that 6909 KEGG metabolic pathways were enriched in all samples. In the rhizosphere group (CK, R1h, Rch, S1h, and Sch treatments), we identified 5321, 5432, 5442, 5442, and 4323 shared KEGG metabolic pathways, respectively, which accounted for 77.02%, 78.65%, 78.77%, 78.77%, and 62.57% of the total, respectively. In the rhizoplane, R1s, Rcs, S1s, and Scs treatments screened 5147, 5059, 5228, and 5328 shared KEGG metabolic pathways, respectively. These accounted for 74.50%, 73.22%, 75.67%, and 77.17% of the total, respectively. In the endosphere bacteria, R1r, Rcr, S1r, and Scr treatments had 5083, 4943, 5289, and 5906 common KEGG metabolic pathways, respectively, which accounted for 73.57%, 71.54%, 76.55%, and 85.48% of the total, respectively. We screened five functions at the L1 level: cellular process, environmental information process, organism system, genetic information process, and metabolism. Among these, genes with metabolic functions were the most abundant (**Figure 8**), followed by those associated with genetic information process and environmental information process. This shows that our treatment affected the survival, reproduction, and metabolism of root microorganisms.

**Figure 9:**
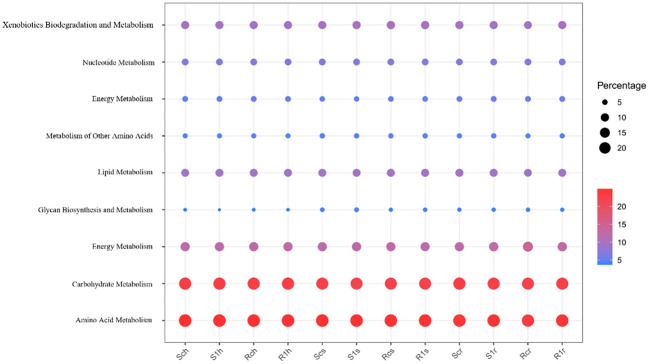
9 functions account for in rhizosphere, rhizoplane and endosphere.

Moreover, we performed an L2 level screening of metabolism. Nine functions with the highest expression levels were analyzed. Carbohydrate and amino acid metabolism accounted for the highest proportion (**Figure 9**). Further comparative analysis found that in the root surface region, carbohydrate metabolism and amino acid metabolism of the susceptible varieties were significantly reduced after inoculation, while there was no significant change in the resistant varieties. This shows that the metabolic capacity of root microorganisms in susceptible varieties is weakened after inoculation with clubroot fungi and that the microorganisms are inhibited in competition with pathogenic bacteria. The root microorganisms of disease-resistant varieties maintain the intensity of their metabolic capabilities, so they occupy an advantageous position in competition with pathogenic bacteria, inhibit the growth and development of pathogenic bacteria, and thus show resistance.

At the same time, we perform L2 level screening on metabolism. And the 9 functions with the highest expression levels were analyzed. It was found that carbohydrate metabolism and amino acid metabolism accounted for the highest proportions (**Figure 9**). Further comparative analysis found that in the root surface region, the carbohydrate metabolism and amino acid metabolism of the susceptible varieties were significantly reduced after inoculation, while there was no significant change in the resistant varieties. This shows that the metabolic capacity of root microorganisms in susceptible varieties is weakened after inoculation with clubroot fungi, and they are inhibited in competition with pathogenic bacteria. The root microorganisms of disease-resistant varieties maintain the intensity of their metabolic capabilities, so they occupy an advantageous position in the competition with pathogenic bacteria, inhibit the growth and development of pathogenic bacteria, and thus show resistance.

### Influence of root secretions on clubroot

We conducted a study to examine the effect of root secretions from resistant and susceptible varieties on the development of *P. brassicae* in cabbage (**Table 1**). Our findings revealed that root secretions of susceptible varieties could increase the disease index even in the absence of bacterial infestation. Specifically, the disease index increased from 49.00 in the control group to 77.78. However, when the cabbage plants were infested with *P. brassicae*, the root secretions of both resistant and susceptible varieties, which contained *P. brassicae* control substances, reduced the incidence rate from 100% to 62.29% and the disease index from 77.78% to 21.43%. Additionally, the root secretions of disease-resistant varieties decreased the incidence rate by 66.66% and the disease index by 38.89%. Interestingly, we observed that the root flora structure remained more stable under *P. brassicae* infestation. This suggests that the root secretions of disease-resistant varieties may enhance disease resistance by stabilizing the root flora structure and inhibiting the propagation of *P. brassicae*

**Table 1:** Effects of different root exudates on club root.

Treatments	CK	Sc	S1	Rc	R1
Incidence of disease	100.00a	100.00a	62.29c	77.78b	33.34d
Disease Index	53.33b	77.78a	21.43d	45.03c	11.11e

Note: a to e represent significance.

## Discussion

### Disease-resistant varieties stabilize root microflora structure

Root microorganisms include rhizosphere, rhizoplane, and endosphere microorganisms. Microbial abundance in the three root chambers showed a gradient (Tao et al., 2022), and their colonization was affected by cultivar factors (Rodriguez et al., 2019). Recent research has shown that microorganisms in initially homogenized soil rapidly differentiate into microbial communities with different functions after planting different varieties of plants (Pereira et al., 2023). In this study, bacterial communities with a relative abundance greater than 1% in the rhizosphere, rhizoplane, and endosphere were significantly different between the resistant and susceptible varieties (**Figure 3**). The bacterial diversity was higher in the rhizoplane and endosphere than in the rhizosphere. After inoculation with *P. brassicae*, the proportion of significantly different bacteria in the rhizosphere, rhizoplane, and endosphere increased, but the proportion of significantly different bacteria in susceptible varieties was higher than 2.6%, and the proportion of significantly different bacteria in resistant varieties was lower than 1.3% (**Supplementary Tables 2**-**4**). This shows that when subjected to *P. brassicae* stress, the abundance of some microorganisms in susceptible varieties changes significantly, while resistant varieties maintain the stability of the microbial community. Bakker et al. (2018) and Oliva et al. (2020) also pointed out that resistant varieties can specifically enrich beneficial microorganisms and make the root microbial structure more stable. Zhang et al. (2023) proposed the “entropy” theory of agriculture: the lower the entropy, the more orderly the interactions between root microorganisms and the stronger the plant’s stress resistance. In a study of microorganisms on rose powdery mildew, Zhao et al. (2020) found that resistant varieties showed resistance traits by recruiting beneficial microorganisms, such as M7SB 41 (*Seimatosporium* sp.), to maintain microbial community functions. In this study, after Chinese cabbage encountered *P. brassicae* stress, susceptible varieties could not regulate the microbial community, or the intensity of regulation was not as high as the rate at which *P. brassicae* occupied the ecological niche. This leads to major changes in the structure of the root flora, which cannot maintain the stability of the root ecosystem, and the entropy value is high, making plants susceptible to disease. Disease-resistant varieties strongly regulate microbial communities, it can inhibit the development of *P. brassicae* and recruit other beneficial microorganisms to maintain microbial diversity and community functions. The entropy value was low, and the plants showed resistance.

### Response of root secretions to microorganisms

Secretion ability is one of the most important metabolic characteristics of plant roots. About 5–21% of the photosynthetically fixed carbon is transferred to the plant rhizosphere through root exudates (Lynch and Whipps, 1990; Nguyen, 2003; Derrien et al., 2004). The type and quantity of root exudates are affected by plant species, growth and development stages, and biotic and abiotic factors (Jones et al., 2004), containing a large number of high molecular weight compounds (such as polysaccharides and proteins), primary metabolites (such as amino acids, organic acids, and sugars), and more than 100,000 secondary metabolites (such as flavonoids, coumarins, hormones, and alkaloids) (Bais et al., 2004; Badri and Vivanco, 2009), whilst the exudates mediate various plant rhizosphere interactions and plays a key role in improving soil structural properties, plant mineral nutrient utilization, and responses to biotic and abiotic stresses (Bais et al., 2006; Yuan et al., 2018; Liu et al., 2020). Zhuang et al. (2020) found that the root exudates of mangroves screen bacterial and fungal communities in the rhizosphere, allowing bacteria beneficial to their growth to take root in the rhizosphere. Root exudates also mediate the toxicity of antibiotics to microorganisms, and antibiotics in the environment shaped by root exudates can change the composition of bacterial colony structures and their functional properties (Tong et al., 2020). In addition, the effects of the root exudates of varieties with different resistance levels were also different. The contents of free amino acids and organic acids in the root exudates of banana-resistant varieties were higher than those of susceptible varieties. The contents of acetic acid and proline are 3.7 times and 2.4 times that of susceptible varieties, respectively, and the root exudates can significantly inhibit the germination of *Fusarium oxysporum* spores (Gan et al., 2020). Zhang et al. (2020) research showed that the root exudates of black shank resistant and susceptible tobacco varieties include different organic acids, alkaloids, fatty acids, and esters. The contents of phenylpropanoid, salicylic acid, fatty acids, 6-hydroxycaproic acid, and hydrogenated jasmonic acid esters in resistant varieties were higher than those in susceptible varieties. Similarly, the root exudates of resistant pepper cultivars can inhibit the hatching and development of root-knot nematodes, whereas those of susceptible cultivars can promote cyst hatching (Çetintaş and Qadir, 2015). Our study also found that Chinese cabbage root exudates play a role in clubroot stress. Root exudates from susceptible varieties increased the disease index, whereas root exudates from resistant varieties decreased the incidence and disease index (**Supplementary Table 4**). This shows that the root exudates of resistant varieties can inhibit the development of clubroot disease to a certain extent. Interestingly, we found that the root exudates of susceptible varieties (S1E) after inoculation with bacteria could also reduce the incidence and disease index, but the degree was not as good as that of the root exudates of resistant varieties (R1E) after inoculation with bacteria. This shows that susceptible varieties have resistance to *P. brassicae*, whereas resistant varieties have other means to suppress *P. brassicae* in addition to autoimmunity. It is possible that resistant varieties also enrich beneficial microorganisms through root exudates to inhibit the growth of *P. brassicae*, thereby greatly reducing the incidence and disease index of clubroot.

### Influence of microorganisms on plant resistance

Root microorganisms play important roles in plant growth and development (Salas-Gonzalez et al., 2021; Van der Heijden et al., 2016). Bais et al. (2006) found that up to 40% of the carbon fixed by plants is released into the rhizosphere through roots, indicating that plants play an active role in shaping the microbial structure of the root system. Recent studies have shown that *Bacillus*, *Pseudomonadaceae*, *Burkholderiaceae*, *Xanthomonas*, and *Actinobacteria* are beneficial for plants (Mauchline and Malone, 2017; Zhao et al., 2016). Among these, *Bacillus* spp. are highly resistant. It has strong resistance to toxic chemicals, such as high temperatures, ultraviolet rays, dryness, and radiation. It is an antagonistic bacterium against a variety of pathogenic microorganisms, and it has a significant inhibitory effect on many bacteria and fungi (Chen et al., 2020). In this study, *Bacillus* did not develop into the dominant bacteria under *P. brassicae* stress, and there was no correlation between the incidence and disease index of clubroot (**Figures 3**, **6**). *Sphingomonas* contributes to the biodegradation and synthesis of aromatic compounds, and it has a bioremediation effect in salt-stressed soils (Liang and Lloyd-Jones, 2010). It can degrade aromatic compounds, such as naphthalene, biphenyl, and phenols in soil (Song et al., 2021). Further, the bacterium can also interact with plants, reducing the proline content of seedlings and roots and increasing the activities of SOD, POD, CAT, and APX (Demidchik, 2015; Khan and Khan, 2017). In this study, *Sphingomonas* was negatively correlated with *P. brassicae* spores and disease index (**Figure 6**), which may be due to the enhancement of plant resistance by increasing the antioxidant capacity of plant roots. *Altererythrobacter* is found in the rhizosphere and in seawater, and it plays an important role in plant salt tolerance (Lee and Whang, 2021; Li et al., 2021). In this study, *Altererythrobacter* was also negatively correlated with *P. brassicae* spores and the disease index (**Figure 6**), which may be involved in inhibiting the occurrence of clubroot. Many studies have shown that *Pseudomonas* can inhibit the growth of bacterial blight in rice (Hofer, 2021). In our study, *Pseudomonas* showed a significant positive correlation with *P. brassicae* and disease index (**Figure 6**). It may be that *Pseudomonas* increases root colonization in response to biotic stress but does not have an inhibitory effect on *P. brassicae*. We also found that the relative abundance of *Flavobacterium* was high in the rhizosphere, rhizoplane, and endosphere of resistant and susceptible varieties, but its correlation with *P. brassicae* spores and the disease index was not significant. We speculate that this may be related to the fact that microbial function does not involve resistance to pathogenic bacteria but is related to plant growth and development.

## Conclusion

Under *P. brassicae* stress, resistant varieties maintain root microbial diversity and microbial community functions through specific root exudates, enriching the genera *Flavobacterium* and *Sphingomonas*.

## Data availability statement

The original contributions presented in the study are publicly available. This data can be found here: https://www.ncbi.nlm.nih.gov/sra/PRJNA1082055.

## Author contributions

TF: Conceptualization, Data curation, Formal analysis, Software, Writing – original draft. XH: Investigation, Writing – review & editing. YY: Methodology, Project administration, Supervision, Writing – review & editing.

## Footnotes

1
https://www.fao.org/faostat/en/#data/QCL

## Supplementary material

Supplementary Figure 1 Phenotypic characterization of Shangpin and 83-1 after inoculation with *P. brassicae*.

Supplementary Table 1 sample tags distribution

Supplementary Table 2 Comparison of the number of different bacteria in rhizosphere among different groups.

Supplementary Table 3 Comparison of the number of different bacteria in rhizoplane among different groups.

Supplementary Table 4 Comparison of the number of different endophytic bacteria among different groups.
