# Supplementary material for: Disease-resistant varieties of Chinese cabbage (Brassica rapa L. ssp. pekinensis) inhibit Plasmodiophora brassicae infestation by stabilising root flora structure
Source: Front Plant Sci. 2024 Mar 5;15:1328845. doi: 10.3389/fpls.2024.1328845 (PMC10950205; doi:10.3389/fpls.2024.1328845)
Supplement: Supplementary Figure 1 — Phenotypic characterization of Shangpin and 83-1 after inoculation with P. brassicae. [file DataSheet_1.docx]

**Supplementary information**


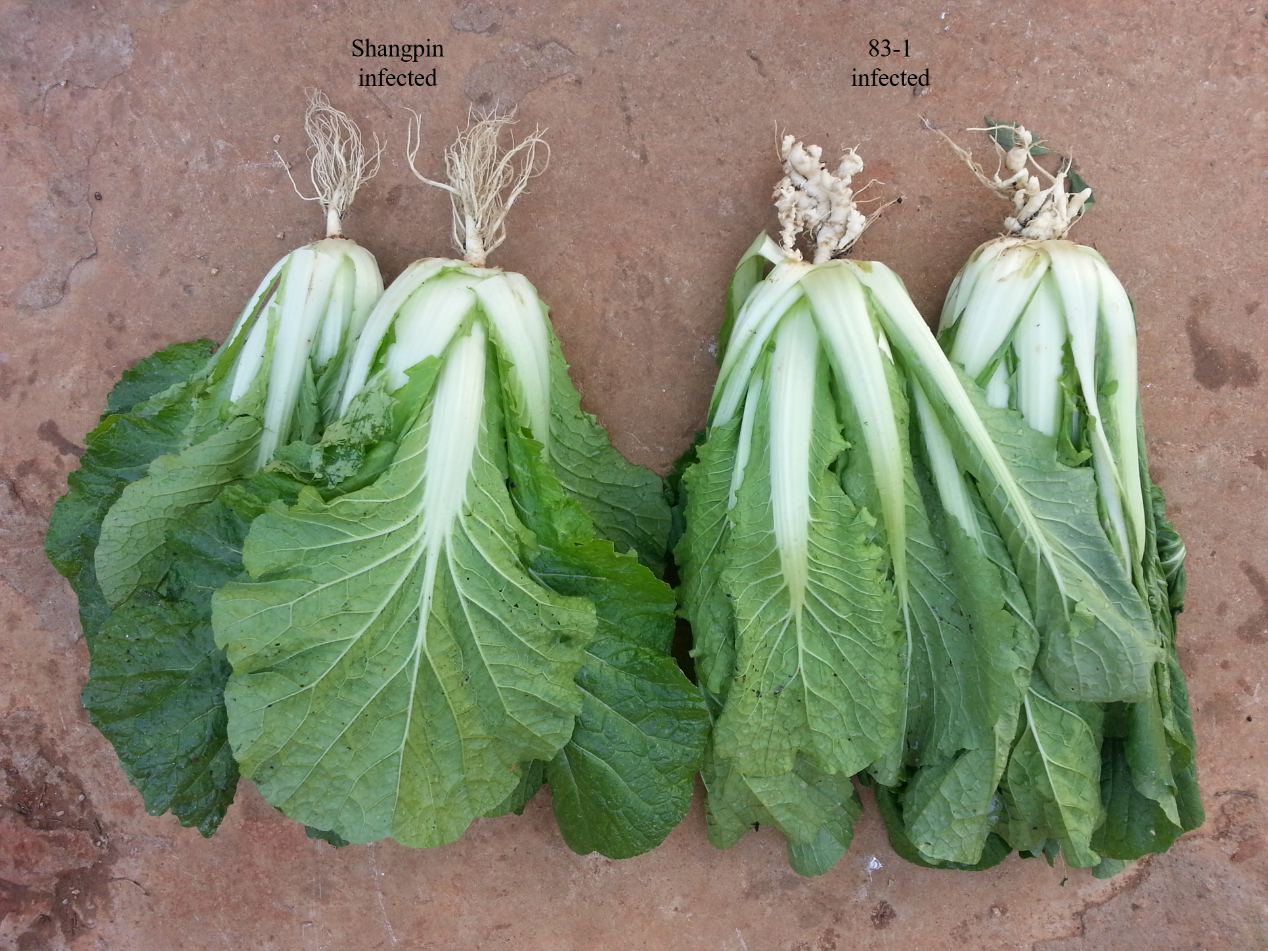


Fig. S1 **Phenotypic characterization of Shangpin and 83-1 after inoculation with** *P. brassicae* **.**

Table S1 sample tags distribution

| Sample ID | clean tags | valid tags | valid percent | valid minLength | valid meanLength | valid maxLength | subsample depth | OTU counts | Total OTUs |
| --- | --- | --- | --- | --- | --- | --- | --- | --- | --- |
| CK.1 | 59330 | 50465 | 0.8506 | 229 | 413.56 | 451 | 36298 | 3355 | 7863 |
| CK.2 | 59942 | 50767 | 0.8469 | 229 | 413.7 | 452 | 36298 | 3323 | 7863 |
| CK.3 | 61271 | 53985 | 0.8811 | 229 | 413.91 | 449 | 36298 | 3364 | 7863 |
| CK.4 | 58901 | 48457 | 0.8227 | 229 | 413.6 | 449 | 36298 | 3361 | 7863 |
| CK.5 | 58591 | 46377 | 0.7915 | 232 | 413.82 | 449 | 36298 | 3282 | 7863 |
| R1h.1 | 58304 | 47902 | 0.8216 | 236 | 414.36 | 449 | 36298 | 3420 | 7863 |
| R1h.2 | 58029 | 47890 | 0.8253 | 236 | 414.16 | 449 | 36298 | 3452 | 7863 |
| R1h.3 | 61453 | 50507 | 0.8219 | 230 | 414.55 | 449 | 36298 | 3462 | 7863 |
| R1h.4 | 61671 | 49322 | 0.7998 | 230 | 414.11 | 451 | 36298 | 3437 | 7863 |
| R1h.5 | 59043 | 48902 | 0.8282 | 229 | 414.55 | 451 | 36298 | 3453 | 7863 |
| R1r.1 | 61747 | 54815 | 0.8877 | 256 | 416.39 | 449 | 36298 | 1365 | 7863 |
| R1r.2 | 58510 | 54826 | 0.937 | 229 | 416.32 | 445 | 36298 | 1393 | 7863 |
| R1r.3 | 59116 | 55062 | 0.9314 | 230 | 416.56 | 457 | 36298 | 1444 | 7863 |
| R1r.4 | 60003 | 54783 | 0.913 | 229 | 417.04 | 442 | 36298 | 1334 | 7863 |
| R1r.5 | 59473 | 53335 | 0.8968 | 229 | 416.93 | 441 | 36298 | 1544 | 7863 |
| R1s.1 | 58963 | 51649 | 0.876 | 230 | 417.87 | 441 | 36298 | 1907 | 7863 |
| R1s.2 | 60108 | 54560 | 0.9077 | 259 | 420.02 | 441 | 36298 | 1768 | 7863 |
| R1s.3 | 61305 | 56278 | 0.918 | 229 | 419.34 | 457 | 36298 | 1807 | 7863 |
| R1s.4 | 59893 | 53207 | 0.8884 | 256 | 418.7 | 459 | 36298 | 1756 | 7863 |
| R1s.5 | 58288 | 53409 | 0.9163 | 256 | 419.58 | 442 | 36298 | 1791 | 7863 |
| Rch.1 | 61390 | 51134 | 0.8329 | 259 | 414.05 | 449 | 36298 | 3304 | 7863 |
| Rch.2 | 59631 | 48440 | 0.8123 | 230 | 414.98 | 449 | 36298 | 3444 | 7863 |
| Rch.3 | 60503 | 50845 | 0.8404 | 229 | 414.62 | 449 | 36298 | 3376 | 7863 |
| Rch.4 | 61216 | 50538 | 0.8256 | 220 | 414.75 | 449 | 36298 | 3436 | 7863 |
| Rch.5 | 60032 | 50595 | 0.8428 | 229 | 414.5 | 450 | 36298 | 3435 | 7863 |
| Rcr.1 | 60716 | 58165 | 0.958 | 228 | 409.93 | 456 | 36298 | 1102 | 7863 |
| Rcr.2 | 61660 | 53577 | 0.8689 | 227 | 416.05 | 449 | 36298 | 2027 | 7863 |
| Rcr.3 | 60375 | 56281 | 0.9322 | 242 | 415.78 | 442 | 36298 | 1459 | 7863 |
| Rcr.4 | 61293 | 56593 | 0.9233 | 228 | 415.29 | 442 | 36298 | 1274 | 7863 |
| Rcr.5 | 60460 | 55956 | 0.9255 | 256 | 416.29 | 457 | 36298 | 1337 | 7863 |
| Rcs.1 | 58992 | 52165 | 0.8843 | 229 | 415.15 | 449 | 36298 | 2634 | 7863 |
| Rcs.2 | 59788 | 55569 | 0.9294 | 228 | 417.23 | 455 | 36298 | 2429 | 7863 |
| Rcs.3 | 59625 | 55430 | 0.9296 | 229 | 419.3 | 449 | 36298 | 2043 | 7863 |
| Rcs.4 | 60787 | 55101 | 0.9065 | 228 | 418.59 | 446 | 36298 | 2110 | 7863 |
| Rcs.5 | 58088 | 49773 | 0.8569 | 236 | 419.19 | 448 | 36298 | 2036 | 7863 |
| S1h.1 | 61036 | 51722 | 0.8474 | 229 | 414.56 | 449 | 36298 | 3586 | 7863 |
| S1h.2 | 59192 | 48823 | 0.8248 | 229 | 414.58 | 460 | 36298 | 3499 | 7863 |
| S1h.3 | 60373 | 49367 | 0.8177 | 259 | 414.07 | 449 | 36298 | 3437 | 7863 |
| S1h.4 | 59780 | 50489 | 0.8446 | 229 | 413.85 | 451 | 36298 | 3493 | 7863 |
| S1h.5 | 61748 | 51535 | 0.8346 | 228 | 414.34 | 449 | 36298 | 3375 | 7863 |
| S1r.1 | 58809 | 52558 | 0.8937 | 229 | 417.27 | 441 | 36298 | 1486 | 7863 |
| S1r.2 | 59247 | 53065 | 0.8957 | 229 | 418.31 | 442 | 36298 | 1445 | 7863 |
| S1r.3 | 56883 | 51614 | 0.9074 | 229 | 414.39 | 441 | 36298 | 1500 | 7863 |
| S1r.4 | 59602 | 52619 | 0.8828 | 229 | 417.91 | 442 | 36298 | 1356 | 7863 |
| S1r.5 | 57309 | 51215 | 0.8937 | 224 | 419.4 | 447 | 36298 | 1319 | 7863 |
| S1s.1 | 58648 | 49948 | 0.8517 | 226 | 418.77 | 453 | 36298 | 1617 | 7863 |
| S1s.2 | 59264 | 53820 | 0.9081 | 228 | 417.21 | 442 | 36298 | 1832 | 7863 |
| S1s.3 | 60802 | 51728 | 0.8508 | 229 | 418.54 | 443 | 36298 | 1804 | 7863 |
| S1s.4 | 61300 | 52064 | 0.8493 | 229 | 418.62 | 442 | 36298 | 1947 | 7863 |
| S1s.5 | 59504 | 50064 | 0.8414 | 229 | 417.51 | 441 | 36298 | 1813 | 7863 |
| Sch.1 | 56204 | 46483 | 0.827 | 229 | 413.48 | 449 | 36298 | 3338 | 7863 |
| Sch.2 | 55269 | 45373 | 0.8209 | 229 | 413.98 | 451 | 36298 | 3378 | 7863 |
| Sch.3 | 59320 | 51336 | 0.8654 | 229 | 414.99 | 452 | 36298 | 3352 | 7863 |
| Sch.4 | 59230 | 46981 | 0.7932 | 236 | 414.8 | 451 | 36298 | 3300 | 7863 |
| Sch.5 | 58661 | 47370 | 0.8075 | 230 | 414.76 | 451 | 36298 | 3373 | 7863 |
| Scr.1 | 60178 | 55395 | 0.9205 | 229 | 417.56 | 460 | 36298 | 1227 | 7863 |
| Scr.2 | 60576 | 55693 | 0.9194 | 230 | 414.81 | 443 | 36298 | 1240 | 7863 |
| Scr.3 | 59147 | 54875 | 0.9278 | 229 | 417.33 | 458 | 36298 | 1328 | 7863 |
| Scr.4 | 58994 | 53512 | 0.9071 | 228 | 415.99 | 444 | 36298 | 1383 | 7863 |
| Scr.5 | 59170 | 54597 | 0.9227 | 229 | 414.48 | 449 | 36298 | 1230 | 7863 |
| Scs.1 | 60109 | 53638 | 0.8923 | 259 | 418.67 | 458 | 36298 | 1760 | 7863 |
| Scs.2 | 60020 | 54687 | 0.9111 | 229 | 417.98 | 450 | 36298 | 1678 | 7863 |
| Scs.3 | 60165 | 54600 | 0.9075 | 229 | 418.85 | 441 | 36298 | 1747 | 7863 |
| Scs.4 | 60663 | 54902 | 0.905 | 229 | 418.59 | 443 | 36298 | 1714 | 7863 |
| Scs.5 | 58628 | 52912 | 0.9025 | 229 | 418.1 | 456 | 36298 | 1773 | 7863 |

**Table S2. Comparison of the number of different bacteria in rhizosphere among different groups**.

| Group | Difficent level | Total number | Number | Proportion(％) |
| --- | --- | --- | --- | --- |
| R1 and S1 | p<0.05 | 668 | 42 | 6.29 |
|  | p<0.01 |  | 12 | 1.8 |
| R1 and Rc | p<0.05 | 684 | 40 | 5.85 |
|  | p<0.01 |  | 6 | 0.88 |
| Rc and Sc | p<0.05 | 701 | 52 | 7.42 |
|  | p<0.01 |  | 10 | 1.43 |
| S1 and Sc | p<0.05 | 689 | 54 | 7.84 |
|  | p<0.01 |  | 18 | 2.61 |

**Table S3. Comparison of the number of different bacteria in rhizoplane among different groups**.

| Group | Difficent level | Total number | Number | Proportion(％) |
| --- | --- | --- | --- | --- |
| R1 and S1 | p<0.05 | 598 | 127 | 21.24 |
|  | p<0.01 |  | 70 | 11.71 |
| R1 and Rc | p<0.05 | 619 | 48 | 7.75 |
|  | p<0.01 |  | 8 | 1.29 |
| Rc and Sc | p<0.05 | 596 | 92 | 15.44 |
|  | p<0.01 |  | 33 | 5.54 |
| S1 and Sc | p<0.05 | 556 | 133 | 23.92 |
|  | p<0.01 |  | 68 | 12.23 |
| R1 and S1 | p<0.05 | 598 | 127 | 21.24 |
|  | p<0.01 |  | 70 | 11.71 |

**Table S4. Comparison of the number of** **different endophytic bacteria among different groups.**

| Group | Difficent level | Total number | Number | Proportion(％) |
| --- | --- | --- | --- | --- |
| R1 and S1 | p<0.05 | 529 | 25 | 4.73 |
|  | p<0.01 |  | 10 | 1.89 |
| R1 and Rc | p<0.05 | 544 | 26 | 4.78 |
|  | p<0.01 |  | 7 | 1.29 |
| Rc and Sc | p<0.05 | 542 | 36 | 6.64 |
|  | p<0.01 |  | 6 | 1.11 |
| S1 and Sc | p<0.05 | 526 | 47 | 8.94 |
|  | p<0.01 |  | 14 | 2.66 |
| R1 and S1 | p<0.05 | 529 | 25 | 4.73 |
|  | p<0.01 |  | 10 | 1.89 |
